# Spectrum of BRCA1/2 variants in 940 patients from Argentina including novel, deleterious and recurrent germline mutations: impact on healthcare and clinical practice

**DOI:** 10.18632/oncotarget.10814

**Published:** 2016-07-24

**Authors:** Angela Rosaria Solano, Florencia Cecilia Cardoso, Vanesa Romano, Florencia Perazzo, Carlos Bas, Gonzalo Recondo, Francisco Bernardo Santillan, Eduardo Gonzalez, Eduardo Abalo, María Viniegra, José Davalos Michel, Lina María Nuñez, Cristina Maria Noblia, Ignacio Mc Lean, Enrique Diaz Canton, Reinaldo Daniel Chacon, Gustavo Cortese, Eduardo Beccar Varela, Martín Greco, María Laura Barrientos, Silvia Adela Avila, Hector Daniel Vuotto, Antonio Lorusso, Ernesto Jorge Podesta, Oscar Gaspar Mando

**Affiliations:** ^1^ Genotipificación y Cáncer Hereditario, Centro de Educación Médicae Investigaciones Clínicas “Norberto Quirno” (CEMIC), Buenos Aires, Argentina; ^2^ Instituto de Investigaciones Bioquímicas (INBIOMED), Facultad de Medicina, Universidad de Buenos Aires-CONICET, Buenos Aires, Argentina; ^3^ Servicio de Oncología, Centro de Educación Médicae Investigaciones Clínicas “Norberto Quirno” (CEMIC), Buenos Aires, Argentina; ^4^ Hospital Alemán, Buenos Aires, Argentina; ^5^ Sanatorio Mater Dei, Buenos Aires, Argentina; ^6^ Mastología, Instituto de Oncología “Angel H. Roffo”, Universidad de Buenos Aires, Buenos Aires, Argentina; ^7^ Instituto Universitario CEMIC, Buenos Aires, Argentina; ^8^ Centro de Estudios de Estado y Sociedad (CEDES), Buenos Aires, Argentina; ^9^ Facultad de Medicina, Universidad de Buenos Aires, Buenos Aires, Argentina; ^10^ Genética, Centro de Educación Médicae Investigaciones Clínicas “Norberto Quirno” (CEMIC), Buenos Aires, Argentina; ^11^ Centro Mamario, Hospital Universitario Austral, Buenos Aires, Argentina; ^12^ Patología Mamaria, Hospital de Clínicas “José de San Martin”, Buenos Aires, Argentina; ^13^ Hospital Regional Victor M. Sanguinetti, Comodoro Rivadavia, Chubut, Escalante, Argentina; ^14^ Hospital Provincial de Neuquén, Neuquén, Argentina; ^15^ Liga Argentina de Lucha contra el Cancer (LALCEC), Buenos Aires, Argentina; ^16^ DAM, Centro de Educación Médicae Investigaciones Clínicas “Norberto Quirno” (CEMIC), Buenos Aires, Argentina

**Keywords:** BRCA1/2 spectrum, BRCA1/2 recurrent mutations, Latin American BRCA1/2 mutations, hispanic panel, genetic testing policy

## Abstract

*BRCA1/2* mutations in Latin America are scarcely documented and in serious need of knowledge about the spectrum of BRCA pathogenic variants, information which may alter clinical practice and subsequently improve patient outcome. In addition, the search for data on testing policies in different regions constitutes a fundamental strength for the present study, which analyzes *BRCA1/2* gene sequences and large rearrangements in 940 probands with familial and/or personal history of breast/ovary cancer (BOC). In non-mutated DNA samples, Multiplex Ligation-dependent Probe Amplification assays (MLPA) were used for the analysis of large rearrangements.

Our studies detected 179 deleterious mutations out of 940 (19.04%) probands, including 5 large rearrangements and 22 novel mutations. The recurrent mutations accounted for 15.08% of the total and only 2.87% of the probands analyzed, very different from a Hispanic panel previously described.

In conclusion: a) this first comprehensive description of the spectrum in BRCA1/2 sheds light on the low frequency of recurrent mutations; b) this information is key in clinical practice to select adequate sequencing studies in our population, subsequently improve patient outcome and prevent damage associated to false normal reports resulting from the use of invalid population panels; c) panels of mutations from other populations should be cautiously validated before imported, even those of apparently similar origin, a concept to be considered beyond significance in Argentina.

## INTRODUCTION

Germline mutations in one of the breast cancer susceptibility genes, *BRCA1* (MIN #113705) or *BRCA2* (MIN#600185), are the major and most widely known risk factors for breast and/or ovarian cancer (BOC) hereditary syndrome (HBOC) [1, 2], although they are present in about 40% of cases with strong family BOC background. HBOC occurs in 5-10% of all BOC cases; in turn, individuals with such inheritance have a 50-80% risk of developing breast cancer and a 30-50% risk of ovarian cancer in their lifetime, while other malignancies such as prostate and pancreatic cancer have been less frequently observed [3, 4]. Furthermore, cancer types such as melanoma and colon have been detected in families with *BRCA2* mutations [3-5].

Since the discovery of *BRCA1* and *BRCA2* genes, thousands of genetic variants with different clinical significance have been described in the Breast Cancer Information Core Database (http://research.nhgri.nih.gov/bic/ access in october 2015), with near 2000 of them being classified as pathogenically responsible for HBOC. The large extension of these genes and the rare hot spot mutations generate a genetic diversity with more than 700 pathogenic mutations described only once.

The frequency of *BRCA1* and *BRCA2* mutation carriers in women with BOC depends on the population analyzed but appears to be similar across ethnicity [6]. However, significant variation has been demonstrated in the spectrum of *BRCA1/2* mutations according to ethnic and/or geographical diversity [7, 8]. Racial mixture in the South American population has been reported in epidemiological and molecular studies [9]. In particular, the Argentine population consists of an admixture of European ancestry -mainly from Spain and Italy- and an Amerindian component in a variable degree which is observed in more than 50% of the population [8, 10, 11].

The epidemiology of HBOC in Argentina has been described in a single report, which reveals racial admixture and clinically relevant genetic variants, including deleterious mutations previously unexplored [8]. Other studies have reported a Hispanic panel [12] including 9 recurrent deleterious mutations which account for 53% of the total, although it should be pointed out that the composition of the so-called Hispanic population analyzed in this publication reflects the Mexican and Central America/Caribbean origin for these probands, completely different from Argentine ethnicity. Big efforts have been made to select a panel of mutations for South American populations such as Peru [13], Mexico [14], Chile [15] and Colombia [12] and they all share a common goal: to lower study costs. Although attention needs to be drawn to the implementation of a mutation panel as a putative screening standard anticipating its impact on health care, “non-mutation detected” results for this panel should be followed by the full sequence of *BRCA1/2*. A panel may become, however, the only analysis in a patient's lifetime, at least in Argentina; in other words, he/she might never be analyzed for the total sequence because of our heterogeneous health insurance system. This secondary effect may prove harmful and confusing for patients and doctors, who may never realize the test performed is practically useless. In addition, limited data on testing outcome and the need for information on testing policies in different regions around the world makes this study all the more relevant. A review has been recently published describing the current knowledge on *BRCA1* and *BRCA2* variants in 4835 women from Latin America, the Caribbean and the Hispanic population in the United States. The study concludes that countries with high prevalence of *BRCA* pathogenic variants may benefit from more aggressive testing strategies, while testing of recurrent variant panels might present a cost-effective solution for improving genetic testing for hereditary cancer in some, but not all countries [16].

These arguments highlight the importance of analyzing *BRCA1/2* gene sequences and large rearrangements, searching for a regional spectrum of variants to contribute to the clinical application of genetic data and the evaluation of HBOC risk in a Hispanic population of different origin. In this context, the present report analyzed 940 probands with HBOC from the Argentine population.

## RESULTS

The analysis of 940 probands revealed 179 (19.04%) deleterious mutations including five large rearrangements: 105 found in the *BRCA1* and 74 in the *BRCA2* gene, all of them detailed in the Supplementary table, with the exception of the novel mutations listed in Table 2. The distribution of the mutations was as follows: a) 105 probands bore a mutation in the *BRCA1* gene, with 8 cases having breast/ovary cancer diagnosis, 11 cases with ovary cancer diagnosis (one is a novel mutation, c.2728C > T, p.Gln910*) and 85 with breast cancer diagnosis, 6 of which were bilateral; b) in 74 probands the mutation was detected in the *BRCA2* gene and include 6 cases with breast/ovary cancer diagnosis (two of them with a novel mutation: c.8754+1G > A and c.8463dupT, p.Ile2822Tyrfs), 2 cases with ovary cancer diagnosis (one is a novel mutation, c.7805+2_7805+3delTA, in intron 16) and the rest with breast cancer diagnosis, 5 female bilateral cases and 3 male cases, one of them bilateral.

**Table 1:** Patients analyzed: Total=940 probands, with a mutation=179, *BRCA1*=105 and *BRCA2*=74

Group	n	Patients with a mutation / Nv (n)	Tumor/s in patients with a mutation (n)
Diagnosed ≤ 40 years Healthy ≤ 40 years	198 135	*BRCA1*: 41 / 4 *BRCA2*: 18 / 6 *BRCA1*: 5 *BRCA2*: 4 / 1	Br(33)/b-Br(5)/Br & Ov(2)/Ov(4)/b-Br & Ov(1) Br(20)/b-Br(2)/Br & Ov(1)/Ov(1) ----- -----
Diagnosed > 40 years Healthy > 40 years	498 95	*BRCA1*: 45 / 3 *BRCA2*: 31 / 5 *BRCA1*: 5 / 1 *BRCA2*: 4 / 1	Br(33)/b-Br(2)/Br & Ov(3)/Ov(7)/b-Ov(1)/b-Br & Ov(2) Br(29)/b-Br(1)/Br & Ov(4)/b-Br & Ov(1)/b-Ov(1) ----- -----
Men diagnosed ≤ 40 years Men diagnosed > 40 years Men Healthy > 40 years	1 12 1	*BRCA1*: 1 *BRCA2*: 0 *BRCA1*: 0 *BRCA2*: 3 *BRCA1*: 0 *BRCA2*: 0 /1	Br & pancreas (1) ----- ----- Br(2)/b-Br(1) ----- -----
Total	940	*BRCA1*: 97 / 8 *BRCA2*: 60 / 14	----- -----

n: number of probands. Nv: novel mutation. Br:breast; Ov: ovary; b-: bilateral

**Table 2:** Novel mutations in *BRCA1*/2detected in 940 probands with personal and/or family history of breast/ovary cancer

ID	Exon/Intron	Mutation	HGVS cDNA	HGVS protein	STOP codon	PH (age)	FH (1st and 2nd degree)
	***BRCA1***						
AB0425	11	*FrameShift*	c.1140delG	p.Gln380=fs	Leu393*	healthy (46)	Br/Ov
AB0020	11	*FrameShift*	c.1502_1505delAATT	p.Lys501=fs	Glu530*	Br (32)	NO
AB0067	11	*FrameShift*	c.2507_2508delAA	p.Glu836Glyfs	Val837*	Br (50)	Br
AB0085	11	*FrameShift*	c.2686delA	p.Ser896Valfs	Leu999*	Br (55)	Br
AB0017	11	*FrameShift*	c.3758_3759delCT	p.Ser1253*	Ser1253*	Br (31)	Br
AB0278	23	*FrameShift*	c.5463_5464insT	p.His1822Serfs	Glu1829*	Br (36)	Br
AB0568	11	*Nonsense*	c.4042G>T	p.Gly1348*	Gly1348*	Br (25)	Br
AB0084	11	*Nonsense*	c.2728C>T	p.Gln910*	Gln910*	Ov (55)	Br, Ov, Co
	*BRCA2*						
AB0364	7	*Splicedefect*	c.517G>T	p.Gly173Cys		Br (48)	Br/Pr
AB0099	9i	*Splicedefect*	c.793+1delG			BR(35)	Br
AB0615	16i	*Splicedefect*	c.7805+2delTA			Ov (43 & 46)	Br/Ov
AB0211	21i	*Splicedefect*	c.8754+1G>A			Br (33)/Ov (36)	Br/Pr
AB0034X	11	*FrameShift*	c.3343delT	p.Ser1115Leufs	Leu1118*	Br (33)	NO
AB0314	11	*FrameShift*	c.4740_4741dupTG	p.Glu1581Valfs	Ser1617*	Br (37)	Br
AB0384	11	*FrameShift*	c.4963delT	p.Tyr1655Thrfs	Leu1669*	healthy (36)	Br/Mel/Pancr
AB0225	11	*FrameShift*	c.5669_5673delTGGCA	p.Met1890Argfs	Leu1897*	Br (52)	Br
AB0098	14	*FrameShift*	c.7110dupA	p.Ser2371Ilefs	Glu2391*	Br (31)	Br
AB0322	14	*FrameShift*	c.7230delT	p.Phe2410Leufs	Val2466*	healthy (41)	Br
AB0392	18	*FrameShift*	c.8021delA^a^	p.Lys2674Argfs	Ile2675*	Br (40)	Br
AB0078U	19	*FrameShift*	c.8463dupT	p.Ile2822Tyrfs	Glu2844*	Br / Ov (54)	NO
AB0048Y	26	*FrameShift*	c.9498delT	p.Val3166=fs	Leu3216*	Healthymale (65)	Br
AB0508	27	*FrameShift*	c.9789_9790delGA	p.Lys3263=fs	Ser3275*	Br (46)	Br

PH: Personal historyof cancer; FH: Family historyof cancer; Br: Breast; Ov: Ovary; Co: colon; Pr: Prostate; Mel: Melanoma; Pancr: Pancreas.

ª: coexistent with BRCA1 c.4484+3A>G also novel.

Age range at first diagnostic: 25-55 years; mean+SD=40.89+9.65 (n =18)

It may be worth highlighting that 230 patients analyzed from a total of 940 had a family history but no personal history of cancer. However, and even if their health status may result from not having inherited the allele with the mutation, they were included in the total patients, as 22 (mean age = 39, range 18-68) out of these 230 were found to be carriers of a mutation (for the non-carriers, *n* = 208, mean age = 41, range = 23-61).

Table 2 lists the 22 novel (at the moment of the detection) deleterious mutations, 8 in *BRCA1* and 14 in *BRCA2,* which represent 12.3% of total mutations. All of them are tier 1, as the resulting stop codon from the frameshift mutation is already associated with a deleterious effect. Table 3 lists a total of 8 novel variants, 2 in *BRCA1* and 6 in *BRCA2,* with probably deleterious effect on the resulting protein, as observed in *in silico* analyses. In both groups, the vast majority had breast cancer diagnosis, with only two cases of ovarian cancer (one with a mutation in each *BRCA* gene, both novel variants) and two cases with both breast and ovary cancers (both with a mutation in *BRCA2)*, all of them in the novel variant group (Table 2).

**Table 3:** Novel variants in B*RCA1/2* with probably deleterious effect from the *in silico* analysis detected in 940 probands with personal and/or family history of breast/ovary cancer

ID	Exon/ Intron	Mutation	HGVS cDNA	HGVS protein	PH (age)	FH (1st and/or 2nd degree)	Align GVGD	PolyPhen	SIFT
	***BRCA1***								
AB0392	14i	Splice defect	c.4484+3A>G ª		Br (40)	Br			
AB0081U	7	Missense	c.341C>G	p.Ser114Cys	Br (45)	Br; Co	Class C0	Possibly damaging	Afect protein function
	***BRCA2***								
AB0376	14	In Frame del	c.7426_7428delGAA	p.Glu2476del	Br (39)	NO			
AB0402	10	Missense	c.1277A>C	p.Lys426Thr	Br (42)	Br	Class C0	Possibly damaging	Afect protein function
AB0185	11	Missense	c.3316A>G	p.Ser1106Gly	Healthy (43)	Br, Gastric	Class C0	Probably damaging	Afect protein function
AB0231	14	Missense	c.7159G>C	p.Ala2387Pro	Healthy (46)	Br, Ov	Class C0	Possibly damaging	Afect protein function
AB0258	18	Missense	c.8038G>A	p.Asp2680Asn	Br (37)	Br	Class C0	Probably damaging	Afect protein function
AB0435	27	Missense	c.9794G>A	p.Cys3265Tyr	Br (50 & 52)	NO	Class C0	Possibly damaging	Afect protein function

PH: Personal historyof cancer; FH: Family historyof cancer; Br: Breast; Ov: Ovary; Co: colon.

ª: coexistent with in BRCA2 c.8021delA, p.Lys2674Argfs -also novel-.

Age range at first diagnostic: 37-50 years; mean+SD=42.17+4.71(n=6)

Recurrent mutations are listed in Table 4, which shows four different deleterious mutations detected 4 or more times in 27 probands, and which represent 2.87% of the total probands analyzed and 15.08% of the patients with a detected mutation. Two additional mutations, the c.5123C > A p.Ala1708Glu in *BRCA1* and c.9026_9030delATCAT p.Tyr3009_His3010fs in *BRCA2* were found in three probands each, increasing frequency of recurrent mutations from 2.87% to 3.52% of the total probands analyzed.

**Table 4:** Recurrent mutations in *BRCA1/2* detected in 940 probands with personal and/or family history of breast/ovary cancer

MUTATION	UNRELATED PROBANDS (% of the total probands)	REPORTED ORIGIN
*BRCA1*		
c.211A>G (p.Arg71Gly)	11 (1.17)	Spanish
c.181T>G (p.Cys61Gly)	6 (0.64)	Italian
		
*BRCA2*		
c.2808_2811delACAA (p.Lys936_Gln937LysGlnfs)	6 (0.64)	French
c.6037A>T (p.Lys2013*)	4 (0.42)	Portuguese / German
Total recurrents	32 (3.4)	-
Total recurrents / total mutated (179) = 17.8%	-	-

Note: The mutation in *BRCA1*: c.4964_4982del19 (p.Ser1655Tyrfs) is to be added in the recurrent mutations panel as it was found five times, including three recent patients (study in progress, see text).

Preliminary results from the sequencing of an additional series of 50 ovary cancer patients (own data in process) showed three instances of the *BRCA1* mutation c.4964_4982delCTGGCCTGACCCCAGAAGA, p.Ser1655_Glu1661?fs. Even if not included in the recurrent panel, this 19bp deletion in our patients appears worth mentioning, as it is listed twice in the Supplementary table and thus renders a 5-time frequency of clinical relevance.

Also worth highlighting, no Ashkenazi mutations were found out of the Ashkenazi ethnicity, as confirmed two detections of the 6174delT (BIC nomenclature) (c.5946_5946delT, p.Ser1982Argfs) with grand/great-grandparents of such ethnic origin.

The large rearrangements listed in Table 5 prove to be rather infrequent mutations in our population, representing only 0.53% (5 of the 940) and all of them corresponding to the *BRCA1* gene, in agreement with patients' ethnic origin as previously described [17, 18].

**Table 5:** Large rearrangements in the *BRCA1* gene detected in 940 probands with personal and/or family history of breast/ovary cancer

ID	Exonsdeleted	HGVS cDNA	PH (age)	FH (1st and 2nd degree)	Nationality / ancestry of families
AB0064X	del 5´UTR thruexon 2	c.1-?_80+?del	Br (35)	Br	Spanish
AB0627	del 5´UTR thruexon 2	c.1-?_80+?del	Ov (40)	Br	Spanish
AB0473	del exons 5 thru 10	c.135-?_670+?del	Br (31)	Br/Gastric Cancer	Slovenian
AB0353	del exons 11 thru 15	c.671-?_4675+?del	Ov (35)	Br	Spanish
AB006H	del exons 15 and 16	c.4485-?_4986+?del	Br (45)	Br	French

PH: Personal history of cancer; FH: Family history of cancer; Br: Breast; Ov: Ovary

## DISCUSSION

This report is to date the first study in our country to summarize the relevant findings of the local experience in the full sequencing of the *BRCA*1/2 genes through the analysis of 940 probands (Supplementary table) in two centers in Argentina.

This study is also the largest on Hispanic families from one country with breast/ovarian cancer in South America, confirming differences from Hispanic families in the United States. In US Hispanic families, recurrent mutations represent 53% of total mutations, which indicates potential for cost-effective ancestry-informed genetic screening Weitzel et al. [12]. In contrast, recurrent mutations in the Hispanic population of South America represent only 15.08% (Table 4), which highlights the importance of clinical genetic strategies adapted to each population's needs and intrinsic genetic characteristics.

One of the important features of our report is the number of probands analyzed. Recently, a very elegant review [16] studying the spectrum of *BRCA1/2* alleles in Latin America and the Caribbean combined 4835 individuals from 13 countries. This review concludes that the Hispanic population of Latin America and the US may benefit from genetic-based cancer prevention options, a strategy that should combine knowledge on hereditary cancer in those populations and improved access to genetic testing. Also in this review, only 10.4% of 167 *BRCA* pathogenic variants identified were shared between US Hispanics and Latin America, a finding regarded as consequence of the limited sample size available for some of the countries. The present study, however, describes results for 940 probands from a single country and still shows the same discrepancy described in the review [16], which clearly proves differences to be associated to real genetic diversity and not limited sample size.

The use of the NGS sequencing technique was crucial in improving our previous report [8], expanding the analysis from about a hundred cases to nearly a thousand probands. Out of these 940 probands, 179 revealed deleterious mutations, which constitutes 19.04%, an expected rate of detection (Supplementary table). Tables 2 and 5 show novel mutations and large rearrangement, respectively, the latter including 5 cases which represent 0.54% of the series.

In the 179 probands analyzed, an expectable correlation was found between cancer type and gene mutation: we found most cases with ovary cancer diagnosis to have mutations in *BRCA1*, 12 cases (one novel) compared with probands with a mutation in *BRCA2*, 2 cases (one novel). The breast and ovary cancer cases were similar for both genes with 7 and 6 cases for *BRCA1* and *BRCA2*, respectively, while the 3 cases of male breast cancer (one bilateral) presented a mutation only in *BRCA2* (Supplementary table).

Large rearrangements are frequent in few ethnic groups [17] and represent only 0.54% of total cases in our study, which is consistent with our patients' ethnic origin detailed in Table 5. These results confirm the differences between the Hispanic families from South America and the United States, where large rearrangements represent 11% of the total. Moreover, the Mexican founder large deletion described by Weitzel et al [12] was not found in our series.

These discrepancies may be due to the differences between the Mexican and Central America/Caribbean origin of these probands, which is remarkably different from Argentine ethnicity, mostly European (Spanish, Italian and German) and Amerindian. Worth pointing out, the Hispanic families from the United States included only 36 probands from South America.

In our population, a single mutation -c.211A > G, Arg71Gly, reported as of Spanish origin- out of the 15 recurrent mutations included in the Hispanic panel [12] has four or more probands; regarding other mutations described in the panel, three were found up to three times in our population: Ala1708Glu (c.5123C > A) and Arg1443* (c.4327C > T) in *BRCA1* and c.9026_9030del5, p.Tyr3009_His3010fs in *BRCA2*. In other words, if the Hispanic panel were applied, these mutations would altogether reach 1.8% of the total probands and 9.5% of the mutated samples. These numbers mean that sequencing the 15 mutations described [12] in 100 probands would render less than 2 patients bearing one of the mutations. Remarkably, the 185delAG (c.66_67delAG, p.Leu22_Glu23LeuValfs) mutation was never found in our series and is included in the Hispanic panel, although it has always been described to occur on the Jewish haplotype [12].

In agreement with the concept of eventual regional variants and/or population heterogeneity, we have previously reported novel mutations in other genes in our population, detected by direct sequencing such as protooncogen *RET* [19], Lynch Syndrome [20], *APC* in Familial Adenomatous Polyposis [21].

Few novel missense variants evaluated *in silico* were found with protein damage prediction programs (Table 3), which heightens the need for cautious interpretation of the variants detected.

**Figure 1:**
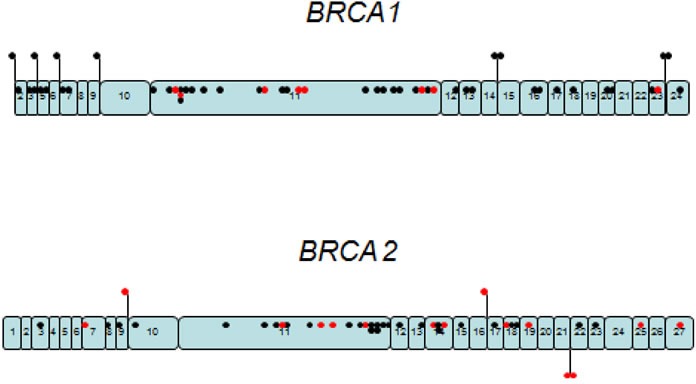
Schematic representation of the gene location for the deleterious mutations detected in the analysis of BRCA1/2 of 940 patients from Argentina Black dots represent the 157 deleterious mutations and the red dots are the 22 novel deleterious mutations, dispersed along each gene.

No hotspots were detected in our population with the variants distributed along both genes (Figure 1), which rules out the possibility of a hot spot zone in the gene or a panel of mutations that might allow the implementation of a low cost initial assay for *BRCA1/2* analysis as attempted in other studies [15].

These striking differences are a warning sign against the import of panels from apparently similar populations. This is a key issue in many aspects: a) clinicians and patients may be misinformed, even in cases with accomplished genetic counseling; b) when a panel is the first analysis, in our health system, insurance may reject further analyses in the same line, i.e. twice the analysis “of the same genes”, which might also be inaccurate, as a full sequencing test is required after a non-mutation has been detected in a panel; c) if health insurance covered both analyses (the panel of mutations and the full sequencing), 97% of the patients analyzed for the recurrent mutations would need full sequencing of *BRCA1/2*, which is not even economically convenient; d) attention needs to be drawn to the correct interpretation of results, as “normal” is considered equivalent to “uncompleted analysis” at two levels: the restricted number of mutations analyzed and the limitations of the *BRCA1/2* analysis itself, a restraining concept for the initial study of a complex genetic study.

Future work will focus on the study of non-BRCA predisposing breast and ovarian cancer genes through multigene panels, although it might be not being easy as the highly conflicts generated by those panels.

This is an important point to be considered in providing the best healthcare possible, mostly in South American countries where the supporting economy is frequently in crisis and low cost studies are attractive. There is a real need for the implementation of highly supported medical care on both ethical and genetic grounds. This will render profits from funds invested in health, mostly in the prevention of high costs for cancer treatments and analyses in hereditary cancer, to be used in prevention (first goal) and early detection.

## CONCLUSIONS

Our results in the sequence analysis of the *BRCA1/2* genes in HBOC probands reveal novel mutations, recurrent mutations and other findings contributing to the knowledge of *BRCA1/2* comprehensive sequence.

We emphasize the following findings: a) no hot spots for *BRCA1/2*, up to date, have been found in patients from our country; b) recurrent mutations have a frequency of 2.87% in the population analyzed and 15.08% in the total mutations, and should therefore, in our opinion, not be implemented for clinical purposes; c) caution must be taken in importing panels for clinical purposes from apparently similar populations; d) panels can make a contribution to shortening studies, but users must be aware of the limitations and alert clinicians and patients, whenever possible.

Further studies and the analysis of the admixture, as a result of successive generations following original immigration, might help understand the origin of inherited mutations in cancer.

## MATERIALS AND METHODS

The study includes 940 patients with ages ranging from 23 to 90 years old, recruited between 2005-2012 from the report [8] and the rest of the patients from January 2013 up to December 2015. They were selected by age at diagnosis before 40 and/or a family history with at least 2 relatives (one of 1st and one of 2nd degree) with breast, ovary or related cancers to *BRCA1/2*, as described in Table 1, which also summarizes the results from the present report. Study eligibility after genetic counseling required signing an informed consent as a result of the routine procedures for genetic analysis (including Ethics Committee approval) at CEMIC (Centro de Educación Médica e Investigaciones Clínicas), which also complies with the Traditional Pretest Counseling for Susceptibility Testing (purpose of testing) described in the American Society of Clinical Oncology Policy Statement Update [22].

As mentioned, a total of 940 samples were analyzed. The first 298 samples were processed with the same methodology used in a previous report [8, 23] and subsequent analysis of the samples was performed by NGS as described below. Genomic DNA was isolated from blood by MagNA Pure® LC instrument with total DNA isolation kit I (Roche Diagnostics). PCR amplification of the regions of interest was done to construct a library with a community panel for *BRCA1* and *BRCA2* using a pool of primers with technology ampliseq™ to amplify exons and exon-intron boundaries of the *BRCA1* and *BRCA2* genes. Sequencing of the amplified regions was performed with the next generation platform Personal Genome Machine® System. As a control, the STR variants of every sample were traced before and intra NGS [24] was used to ensure the identification of the sample and avoid possible processing errors (Genia Laboratory, Zonamerica, Uruguay). The few codifying sequences with low readings were analyzed by Sanger reaction in order to reach 100% coverage, as was the case for every clinically relevant mutation.

Large rearrangements were measured by Multiplex Ligation-dependent Probe Amplification (MLPA) with reagents from MRC-Holland, Amsterdam, ND and software Coffalyser.net was used for data analysis.

Variant nomenclature follows the guidelines for the Human Genome Variation Society (HGVS). Genetic variants detected in a sequence were verified in the Cancer Information Core Internet Website (BIC) as December 2015 (http://research.nhgri.nih.gov/bic/), for clinical importance and to determine whether a variant report (otherwise novel) exists. The effects of those missense mutations which were neither reported nor recorded as clinically unknown (CU) in the BIC were predicted by virtual analyses of functional compatibility for aminoacid changes using software Align-GVGD (http://agvgd.iarc.fr/) [25] and SIFT (http://blocks.fhcrc.org/sift/SIFT.html) [26].

## SUPPLEMENTARY TABLE


