# Supplementary material for: Spectrum of BRCA1/2 variants in 940 patients from Argentina including novel, deleterious and recurrent germline mutations: impact on healthcare and clinical practice
Source: Oncotarget. 2016 Jul 24;8(36):60487–95. doi: 10.18632/oncotarget.10814 (PMC5601155; doi:10.18632/oncotarget.10814)
Supplement: Supplementary file 1 [file oncotarget-08-60487-s001.docx]

| **Table S1**. Deleterious mutations in *BRCA1/2* (n=157) detected in 940 probands with personal and/or family history of breast/ovary cancer. | | | | | | |
| --- | --- | --- | --- | --- | --- | --- |
| Novel deleterious (n=22) mutations are listed in table 3 of the report.  Total mutations detected=179, in *BRCA1* gene n=105 and in *BRCA2* gene n=74 probands | | | | | |  |
| FAMILY ID | Gene | Tumor (age) | BIC Designation | HGVS *DNA level* | FamilyHistory (Y/N) |  |
| AB0064X | BRCA1 | Breast (35) | del 5´UTR thruexon 2 | c.1-?_80+?del | Y |  |
| AB0627 | BRCA1 | Ovary (40) | del 5´UTR thruexon 2 | c.1-?_80+?del | Y |  |
| AB0019D | BRCA1 | Breast (38) | 185delAG | c.68_69delAG | Y |  |
| AB0424 | BRCA1 | bilateral Breast(34&44) | 185delAG | c.68_69delAG | Y |  |
| AB0029E | BRCA1 | Breast (30) | 185delAG | c.68_69delAG | Y |  |
| AB0041F | BRCA1 | Breast (37) | 185delAG | c.68_69delAG | Y |  |
| AB0094G | BRCA1 | Breast (31) | 185delAG | c.68_69delAG | Y |  |
| AB0006I | BRCA1 | Breast (46) | 185delAG | c.68_69delAG | Y |  |
| AB0063I | BRCA1 | Breast (57) | 185delAG | c.68_69delAG | Y |  |
| AB0080I | BRCA1 | Healthy (48) | 185delAG | c.68_69delAG | Y |  |
| AB0003J | BRCA1 | Breast (34) | 185delAG | c.68_69delAG | Y |  |
| AB0004J | BRCA1 | Breast (43) | 185delAG | c.68_69delAG | Y |  |
| AB0033J | BRCA1 | maleBreast(53) | 185delAG | c.68_69delAG | Y |  |
| AB0085J | BRCA1 | Healthy (52) | 185delAG | c.68_69delAG | Y |  |
| AB0086J | BRCA1 | Ovary (58) | 185delAG | c.68_69delAG | Y |  |
| AB0013K | BRCA1 | Breast (43) | 185delAG | c.68_69delAG | Y |  |
| AB0098W | BRCA1 | Breast (50) | 185delAG | c.68_69delAG | Y |  |
| AB0054 | BRCA1 | Breast (37) | 185delAG | c.68_69delAG | Y |  |
| AB0060 | BRCA1 | Breast (40) | 185delAG | c.68_69delAG | Y |  |
| AB0077 | BRCA1 | Ovary (44) | 185delAG | c.68_69delAG | Y |  |
| AB0068 | BRCA1 | Ovary (44) | 185delAG | c.68_69delAG | Y |  |
| AB0076 | BRCA1 | Breast (49) | 185delAG | c.68_69delAG | Y |  |
| AB0081 | BRCA1 | Breast (52) | 185delAG | c.68_69delAG | Y |  |
| AB0088 | BRCA1 | Ovary (60) | 185delAG | c.68_69delAG | Y |  |
| AB0041L | BRCA1 | Breast (48) | 185delAG | c.68_69delAG | Y |  |
| AB0087S | BRCA1 | Breast (49) | 185delAG | c.68_69delAG | Y |  |
| AB0103S | BRCA1 | Breast (42) | 185delAG | c.68_69delAG | Y |  |
| AB0082 | BRCA1 | Breast (34) | 185insA | c.66dupA | Y |  |
| AB0042C | BRCA1 | Healthy (36) | 185insA | c.66dupA | Y |  |
| AB0473 | BRCA1 | Breast (31) | del exons 5 thru 10 | c.135-?_670+?del | N |  |
| AB0594 | BRCA1 | Breast (46) | C61G | c.181T>G | Y |  |
| AB0003E | BRCA1 | Breast (25) | C61G | c.181T>G | Y |  |
| AB0052U | BRCA1 | Breast (35) | C61G | c.181T>G | Y |  |
| AB0088W | BRCA1 | Breast (34) | C61G | c.181T>G | Y |  |
| AB0177 | BRCA1 | bilateral Breast(31) | C61G | c.181T>G | Y |  |
| AB0075 | BRCA1 | Breast (49) | C61G | c.181T>G | Y |  |
| AB0380 | BRCA1 | Ovary (55) | R71G | c.211A>G | Y |  |
| AB0474 | BRCA1 | Breast (44) | R71G | c.211A>G | Y |  |
| AB0540 | BRCA1 | Breast (31) | R71G | c.211A>G | Y |  |
| AB0459 | BRCA1 | Breast (46) | R71G | c.211A>G | Y |  |
| AB0049X | BRCA1 | Breast (50) | R71G | c.211A>G | Y |  |
| AB0052X | BRCA1 | Breast (55) | R71G | c.211A>G | Y |  |
| AB0083X | BRCA1 | Breast (59) | R71G | c.211A>G | Y |  |
| AB0064 | BRCA1 | Breast (43) | R71G | c.211A>G | Y |  |
| AB00P1 | BRCA1 | Breast (50 | R71G | c.211A>G | Y |  |
| AB0602 | BRCA1 | Breast (48), Ovary (50), Rectum+colon(53) | R71G | c.211A>G | Y |  |
| AB0532 | BRCA1 | Breast (33) | R71G | c.211A>G | Y |  |
| AB0001E | BRCA1 | Breast (50) | IVS6-1G>A | c.302-1G>A | Y |  |
| AB0065X | BRCA1 | Breast (42) | E143X | c.427G>T | Y |  |
| AB0046 | BRCA1 | Breast (43) | E143X | c.427G>T | Y |  |
| AB0640 | BRCA1 | Breast (55) | E143X | c.427G>T | Y |  |
| AB0353 | BRCA1 | Ovary (35) | del exons 11 thru 15 | c.671-?_4675+?del | Y |  |
| AB0036 | BRCA1 | Breast&Ovary (46) | 917delTT | c.798_799delTT | Y |  |
| AB0150 | BRCA1 | Healthy (25) | 1186delA | c.1067delA | Y |  |
| AB0333 | BRCA1 | Breast (66) | 1207delA | c.1088delA | Y |  |
| AB0296 | BRCA1 | Breast (37) | 1416delG | c.1297delG | Y |  |
| AB0031K | BRCA1 | Breast (53) | 1479delAG | c.1360_1361delAG | Y |  |
| AB0040 | BRCA1 | Breast (32) | 1629delC | c.1510delC | Y |  |
| AB0340 | BRCA1 | bilateral Breast(49 & 59) + Ovary (59) | Q563X | c.1687C>T | Y |  |
| AB0507 | BRCA1 | Breast&Ovary (41) | 2011insT | c.1892dupT | Y |  |
| AB0028X | BRCA1 | Breast (33) | Y856X | c.2568T>G | Y |  |
| AB0232 | BRCA1 | Breast (43) | E908X | c.2722G>T | Y |  |
| AB0002G | BRCA1 | Breast (52) | 3347delAG | c.3228_3229delAG | Y |  |
| AB0084D | BRCA1 | Healthy (33) | L1086X | c.3257T>G | Y |  |
| AB0041Y | BRCA1 | Healthy (35) | 3477delGT | c.3358_3359delGT | Y |  |
| AB0008 | BRCA1 | Ovary (25) | R1203X | c.3607C>T | Y |  |
| AB0021 | BRCA1 | Breast&Ovary (33) | 3746insA | c.3627dupA | Y |  |
| AB0200 | BRCA1 | Breast (49) | L1230X | c.3689T>G | Y |  |
| AB0311 | BRCA1 | Breast (38) | 3958del5ins4 | c.3839_3843delCTCAGinsAGGC | Y |  |
| AB0550 | BRCA1 | Breast (30) | Q1395X | c.4183C>T | Y |  |
| AB0586 | BRCA1 | Breast (35) | Q1401X | c.4201C>T | Y |  |
| AB0170 | BRCA1 | Breast (40) | R1443X | c.4327C>T | Y |  |
| AB0188 | BRCA1 | Breast (40) | R1443X | c.4327C>T | Y |  |
| AB006H | BRCA1 | Breast (37) | del exons 15 & 16 | c.4485-?_4986+?del | Y |  |
| AB0001X | BRCA1 | Breast (34&58) | 4744delCT | c.4625_4626delCT | Y |  |
| AB0436 | BRCA1 | Breast (67) | 5083del19 | c.4964_4982delCTGGCCTGACCCCAGAAGA | Y |  |
| AB0010X | BRCA1 | Ovary (35) | 5083del19 | c.4964_4982delCTGGCCTGACCCCAGAAGA | Y |  |
| AB0036X | BRCA1 | Breast (37) | 5149del4 | c.5030_5033delCTAA | Y |  |
| AB0079 | BRCA1 | Breast (51) | 5149del4 | c.5030_5033delCTAA | Y |  |
| AB0032E | BRCA1 | Breast (24) | A1708E | c.5123C>A | Y |  |
| AB0071W | BRCA1 | Breast (30) | A1708E | c.5123C>A | Y |  |
| AB0545 | BRCA1 | Breast (30&34) | A1708E | c.5123C>A | Y |  |
| AB0578 | BRCA1 | Healthy (18) | 5382insC | c.5266dupC | Y |  |
| AB0042F | BRCA1 | Ovary (41) | 5382insC | c.5266dupC | Y |  |
| AB0023J | BRCA1 | Healthy (53) | 5382insC | c.5266dupC | Y |  |
| AB0082K | BRCA1 | Breast (41&47) | 5382insC | c.5266dupC | Y |  |
| AB0055 | BRCA1 | Breast (49) | 5382insC | c.5266dupC | Y |  |
| AB0097 | BRCA1 | Breast (38) | 5382insC | c.5266dupC | Y |  |
| AB0038A | BRCA1 | Breast (37 & 38) | 5382insC | c.5266dupC | Y |  |
| AB0092B | BRCA1 | bilateral Breast(60) | 5382insC | c.5266dupC | Y |  |
| AB0458S | BRCA1 | Healthy (57) | 5382insC | c.5266dupC | Y |  |
| AB0003F | BRCA1 | Breast (43) | 5382insC | c.5266dupC | Y |  |
| AB0386 | BRCA1 | Breast (28) | 5382insC | c.5266dupC | N |  |
| AB0396 | BRCA1 | Breast (39)&Ovary (45) | 5382insC | c.5266dupC | Y |  |
| AB0151 | BRCA1 | Healthy (42) | 5382insC | c.5266dupC | Y |  |
| AB0215 | BRCA1 | bilateral Breast&Ovary (68) | 5382insC | c.5266dupC | Y |  |
| AB0096C | BRCA1 | bilateral Breast&ovary (35) | IVS23-1G>A | c.5468-1G>A | Y |  |
| AB0043K | BRCA2 | Breast (41) | E49X | c.145G>T | Y |  |
| AB0412 | BRCA2 | Breast (45) | 886delGT | c.658_659delGT | Y |  |
| AB0008X | BRCA2 | Healthy (24) | 928delT | c.700delT | Y |  |
| AB0326 | BRCA2 | bilateral Breast(39) | 928delT | c.700delT | Y |  |
| AB0244 | BRCA2 | Breast (41) | 3036del4 | c.2808_2811delACAA | Y |  |
| AB0256 | BRCA2 | bilateral Breast(56 & 63)Ovary(56) | 3036del4 | c.2808_2811delACAA | Y |  |
| AB0267 | BRCA2 | Breast (40) | 3036del4 | c.2808_2811delACAA | Y |  |
| AB0137 | BRCA2 | bilateral Breast (37) | 3036del4 | c.2808_2811delACAA | Y |  |
| AB0078 | BRCA2 | Breast (50) | 3036del4 | c.2808_2811delACAA | Y |  |
| AB00P2 | BRCA2 | Breast (54) | 3036del4 | c.2808_2811delACAA | Y |  |
| AB0452 | BRCA2 | Breast (50) | 3492insT | c.3264dupT | Y |  |
| AB0057D | BRCA2 | Male bilateral Breast(60)-actual age: 90y- | E1308X | c.3922G>T | Y |  |
| AB0053Y | BRCA2 | Breast (51) | 4970insTG | c.4740_4741dupTG | Y |  |
| AB0314 | BRCA2 | Breast (41) | 4970insTG | c.4740_4741dupTG | Y |  |
| AB0002E | BRCA2 | Breast&Ovary (52) | 5579insA | c.5351dupA | Y |  |
| AB0160 | BRCA2 | Breast (33) | 5579insA | c.5351dupA | Y |  |
| AB0466 | BRCA2 | Breast (66) | 5579insA | c.5351dupA | Y |  |
| AB0117 | BRCA2 | Breast (50) | S1882X | c.5645C>G | Y |  |
| AB0043E | BRCA2 | bilateral Ovary (65) | S1882X | c.5645C>G | Y |  |
| AB0092 | BRCA2 | Breast (31) | 5909insA | c.5681dupA | Y |  |
| AB0467 | BRCA2 | MaleBreast(82) | 6024delTA | c.5796_5797delTA | Y |  |
| AB0056G | BRCA2 | Breast (45) | 6174delT | c.5946delT | Y |  |
| AB0013I | BRCA2 | Breast (30) | 6174delT | c.5946delT | Y |  |
| AB0037J | BRCA2 | MaleBreast (47)&Pancreas (60) | 6174delT | c.5946delT | Y |  |
| AB0055K | BRCA2 | Healthy (47) | 6174delT | c.5946delT | Y |  |
| AB0042Y | BRCA2 | Breast (47) | 6174delT | c.5946delT | Y |  |
| AB0043 | BRCA2 | Breast (32) | 6174delT | c.5946delT | Y |  |
| AB0047 | BRCA2 | Breast (33) | 6174delT | c.5946delT | Y |  |
| AB0069 | BRCA2 | Breast&Ovary (45) | 6174delT | c.5946delT | Y |  |
| AB0057 | BRCA2 | Breast (39) | 6174delT | c.5946delT | Y |  |
| AB0071 | BRCA2 | Breast (46) | 6174delT | c.5946delT | Y |  |
| AB0074 | BRCA2 | Breast (48) | 6174delT | c.5946delT | Y |  |
| AB0095 | BRCA2 | Breast (36) | 6174delT | c.5946delT | Y |  |
| AB0096 | BRCA2 | Breast (60) | 6174delT | c.5946delT | Y |  |
| AB0065S | BRCA2 | Healthy (32) | 6174delT | c.5946delT | Y |  |
| AB0022A | BRCA2 | Healthy (39) | 6174delT | c.5946delT | Y |  |
| AB0023A | BRCA2 | Breast (52) | 6174delT | c.5946delT | Y |  |
| AB0025F | BRCA2 | Breast (64) | 6174delT | c.5946delT | Y |  |
| AB0091B | BRCA2 | Breast (53) | 6174delT | c.5946delT | Y |  |
| AB0418 | BRCA2 | Breast (36) | 6174delT | c.5946delT | Y |  |
| AB0091W | BRCA2 | Breast (61) | 6174delT | c.5946delT | Y |  |
| AB0504 | BRCA2 | Breast (48) | 6174delT | c.5946delT | Y |  |
| AB0531 | BRCA2 | Breast (28) | 6252insG | c.6024dupG | Y |  |
| AB0575 | BRCA2 | bilateral Breast (44) | K2013X | c.6037A>T | Y |  |
| AB0034 | BRCA2 | Breast (40) | K2013X | c.6037A>T | Y |  |
| AB0222 | BRCA2 | Healthy (48) | K2013X | c.6037A>T | Y |  |
| AB0305 | BRCA2 | Breast (53) | K2013X | c.6037A>T | Y |  |
| AB0505 | BRCA2 | Breast (37) | 6237del4 | c.6209_6212delAAAG | Y |  |
| AB0631 | BRCA2 | Breast (50) | 7047delA | c.6814delA | Y |  |
| AB0557 | BRCA2 | Healthy (68) | R2336P | c.7007G>C | Y |  |
| AB0413 | BRCA2 | Breast (64) | R2394X | c.7180A>T | Y |  |
| AB0421 | BRCA2 | Breast (55) | R2520X | c.7558C>T | Y |  |
| AB0080X | BRCA2 | Healthy (45) | W2619X | c.7857G>A | Y |  |
| AB0031 | BRCA2 | Ovary (38) | D2723H | c.8167G>C | Y |  |
| AB0047X | BRCA2 | Breast&Ovary (54) | 8691insT | c.8463dupT | Y |  |
| AB0085X | BRCA2 | Breast (32) | IVS21-1G>A | c.8755-1G>A | Y |  |
| AB0077E | BRCA2 | Breast (42) | Q2960X | c.8878C>T | Y |  |
| AB0530 | BRCA2 | Healthy (25) | Q2960X | c.8878C>T | Y |  |
| AB0067U | BRCA2 | Breast (52) | 9254del5 | c.9026_9030delATCAT | Y |  |
| AB0265 | BRCA2 | Breast (35) | 9254del5 | c.9026_9030delATCAT | Y |  |
